# Cooperation between coagulase and von willebrand factor binding protein in *Staphylococcus aureus* fibrin pseudocapsule formation

**DOI:** 10.1016/j.bioflm.2024.100233

**Published:** 2024-10-23

**Authors:** Dominique C.S. Evans, Amanda B. Khamas, Alex Payne-Dwyer, Adam J.M. Wollman, Kristian S. Rasmussen, Janne K. Klitgaard, Birgitte Kallipolitis, Mark C. Leake, Rikke L. Meyer

**Affiliations:** a School of Physics, Engineering and Technology, University of York, York, UK; b Interdisciplinary Nanoscience Centre, Aarhus University, Aarhus, Denmark; c Department of Biology, University of York, York, UK; d Department of Biochemistry and Molecular Biology, University of Southern Denmark, Odense, Denmark

**Keywords:** Biofilm, Extracellular matrix, Confocal microscopy, HILO, Monomeric superfolder GFP, SNAP tag, CLIP tag

## Abstract

The major human pathogen *Staphylococcus aureus* forms biofilms comprising of a fibrin network that increases attachment to surfaces and shields bacteria from the immune system. It secretes two coagulases, Coagulase (Coa) and von Willebrand factor binding protein (vWbp), which hijack the host coagulation cascade and trigger the formation of this fibrin clot. However, it is unclear how Coa and vWbp contribute differently to the localisation and dynamics of clot assembly in growing biofilms.

Here, we address this question using high-precision time-resolved confocal microscopy of fluorescent fibrin to establish the spatiotemporal dynamics of fibrin clot formation in functional biofilms. We also use fluorescent fusion proteins to visualise the locations of Coa and vWbp in biofilms using both confocal laser scanning and high resolution highly inclined and laminated optical sheet microscopy. We visualise and quantify the spatiotemporal dynamics of fibrin production during initiation of biofilms in plasma amended with fluorescently labelled fibrinogen.

We find that human serum stimulates coagulase production, and that Coa and vWbp loosely associate to the bacterial cell surface. Coa localises to cell surfaces to produce a surface-attached fibrin pseudocapsule but can diffuse from cells to produce matrix-associated fibrin. vWbp produces matrix-associated fibrin in the absence of Coa, and furthermore accelerates pseudocapsule production when Coa is present. Finally, we observe that fibrin production varies across the biofilm. A sub-population of non-dividing cells does not produce any pseudocapsule but remains within the protective extended fibrin network, which could be important for the persistence of *S. aureus* biofilm infections as antibiotics are more effective against actively growing cells.

Our findings indicate a more cooperative role between Coa and vWbp in building fibrin networks than previously thought, and a bet-hedging cell strategy where some cells produce biofilm matrix while others do not, but instead assume a dormant phenotype that could be associated with antibiotic tolerance.

## Highlights

• Coa and vWbp are loosely associated to bacterial surfaces.
• Coa is essential for fibrin pseudocapsule formation.
• VWbp accelerates pseudocapsule formation in the presence of Coa.
• A sub-population of non-dividing *S. aureus* do not produce pseudocapsules.

## 1 Introduction

Biofilms are bacterial aggregates encased in an extracellular matrix [1], and treating biofilms with antibiotics is notoriously difficult because the biofilm phenotype protects bacteria from the immune system and offers many routes to antibiotic tolerance. These include reduced penetration of certain antibiotics through the matrix [2], and sub-populations of metabolically inactive cells [3] and persister cells [4]. Failure to eradicate a biofilm typically leads to recurrence of the infection shortly after antibiotic treatment stops. *Staphylococcus aureus* is an opportunistic human pathogen that is a commensal in about 30 % of the population, and it causes a number of different infections ranging from skin and soft tissue infections to severe diseases such as endocarditis and osteomyelitis [5]. *S. aureus* is also a leading cause of implant-associated infections, whereby bacteria colonise the surface of an implanted device and form biofilms [6]. When antibiotic treatment fails, surgical removal and replacement of the infected device is often the only solution, which is both costly and associated with medical risks.

A hallmark of *S. aureus* is the ability to coagulate blood and form a fibrin clot. Fibrin is derived from host fibrinogen and is a key component of the extracellular matrix [7]. It forms two concentric structures: a cell surface attached structure and an extended outer network, which act as mechanical barriers against immune attack [8] similar to the stress-absorbing viscoelastic filamentous matrices that many types of cellular systems exhibit [9]. The cell surface attached structure is usually referred to as a pseudocapsule, as the fibrin forms a capsule-like structure that encompasses cells. We refer to the extended outer network as matrix-associated fibrin: fibrin that forms in the intercellular space, rather than in the close vicinity of and associated to the surface of cells. *S. aureus* interacts with host proteins via several surface-associated proteins, including a large family of proteins called Microbial Surface Component Recognising Adhesive Matrix Molecules (MSCRAMMs) [10], and secreted proteins known as Secretable Expanded Repertoire Adhesive Molecules (SERAMs) which also bind to and incorporate host proteins into the extracellular matrix [11]. *S. aureus* secretes two SERAMs, Coagulase (Coa) [12] and von Willebrand factor binding protein (vWbp) [13] which non-proteolytically activate host prothrombin to hijack the host coagulation cascade and form a complex that cleaves fibrinogen, which then polymerises into fibrin fibers [[14], [15], [16]]. Coa and vWbp have homologous *N*-termini with D1-D2 domains that bind prothrombin and fibrinogen [17] but differ in their *C*-termini. Coa has an R domain that consists of multiple repeats of a fibrinogen binding peptide [18], while vWbp has domains that bind von Willebrand factor [13], fibronectin [19], and factor XIII [19]. Hence, the two proteins both trigger fibrin formation, but may do this at different locations or leading to different macromolecular super-structures due to their association with different types of host proteins via the *C*-terminal binding domains.

It is unknown why *S. aureus* expresses two proteins to produce fibrin, and whether these proteins are redundant or carry out different roles, for example to direct fibrin production to a particular location. The prevailing assumption has been that they act independently, with Coa forming the cell surface-associated pseudocapsule and vWbp the matrix-associated fibrin [8]. Fibrin enhances *S. aureus’* virulence, protects the bacteria from the immune system, and aids in adhesion to surfaces. *S. aureus* mutants that lack Coa or vWbp are less virulent, particularly if both coagulases are absent [20]. Inhibiting the active complex with prothrombin also decreases virulence in mice, reduces attachment to surfaces, and increases the ability of leukocytes to clear the infection [21,22]. Furthermore, *S. aureus* that lacks vWbp coagulates blood more slowly than the wildtype, and *S. aureus* lacking Coa coagulates blood even more slowly [23]. Guggenberger et al. (2012) found that *S. aureus* lacking vWbp was unable to form matrix-associated fibrin, and *S. aureus* lacking Coa only partially formed the pseudocapsule, suggesting that Coa primarily produces the pseudocapsule while vWbp forms matrix-associated fibrin [8]. Coa localises within the pseudocapsule [8,20,24] and accumulates at the periphery of abscess lesions [20], while vWbp is distributed throughout abscess lesions and accumulates at the periphery [20]. The Coa-prothrombin complex becomes active immediately, while the vWbp-prothrombin complex activates slowly because it also needs to bind to fibrinogen [15]. vWbp binds to a wider variety of host proteins than Coa, and vWbp has a higher affinity towards surface adsorbed fibrinogen than Coa while they both bind soluble fibrinogen equally [25]. These differences suggest that Coa and vWbp have different roles, such as Coa forming the pseudocapsule as a first line defence against the immune system, and vWbp diffusing further away to attach the biofilm to biological surfaces via other host proteins. However, the mechanisms they use to do so are unknown, and it is not fully established whether their roles overlap.

We used coagulation tests of pelleted cells or supernatants from *S. aureus* batch cultures to determine if Coa and vWbp associate to the bacterial surface, and we subsequently investigated their roles of in *S. aureus* biofilm formation. To determine if each coagulase was responsible for location-specific fibrin formation, we visualised the location of fibrin in wildtype *S. aureus* biofilms, and compared it to knockout mutants lacking Coa, vWbp, or both. We quantified the formation of surface-associated fibrin in the pseudocapsule over time in growing *S. aureus* biofilms to uncover differences in the dynamics of fibrin formation by each coagulase, and how the presence of both coagulases changed this dynamic. The location of each coagulase was visualised by high-precision confocal laser scanning fluorescence (CLSM) and highly inclined and laminated optical sheet (HILO) microscopy of fluorescent fusion proteins of Coa and vWbp.

## 2 Methods

### 2.1 Bacterial strains and growth conditions

All strains of bacteria, plasmids, and primers used are listed in Table 1. Bacteria were cultured in brain heart infusion (BHI, 53,286, Millipore) overnight at 37 °C with 180 rpm shaking. Medium was supplemented with 5–10 % human serum for Coa and vWbp expression, and with 50 % heparin-stabilised human plasma for biofilm growth to allow fibrin formation in the biofilm whilst still providing bacteria with nutrients from BHI for growth. Heparin was used as an anticoagulant when collecting serum and plasma rather than EDTA and citrate because it did not inhibit growth of planktonic *S. aureus*. In experiments studying coagulation, 10 μg/ml chloramphenicol (*Cm*, C0378, Sigma-Aldrich) was added to prevent further production of Coa and vWbp after transferring bacteria to human plasma. Plasma and serum were prepared from blood donated from Aarhus University Hospital. Blood was collected in collection tubes (367,526, BD Vacutainer) and centrifuged at 2000×*g* at 4 °C for 15 min. For plasma separation, blood was collected in tubes that were coated with heparin, whilst for serum separation, tubes were uncoated. After centrifugation, the plasma or serum were pooled, divided into aliquots, and stored at −80 °C. Before use, plasma and serum were thawed in a 37 °C water bath. Calcium and magnesium are important for biofilm structure [26,27], therefore BHI for growing biofilms was further supplemented with 2.1 mM CaCl_2_ (C3881, Sigma-Aldrich) and 0.4 mM MgCl_2_ (31,413, Sigma-Aldrich) to create modified BHI (mBHI) to raise the levels of calcium and magnesium in BHI to physiological levels when combined with 50 % human plasma.

**Table 1:** All bacteria strains, plasmids, and primers used throughout this study. Annealing parts of primers are given in capital letters, and overhangs in lowercase.

Bacterial Strain	Description	Reference
*Staphylococcus aureus* ATCC 29213	Clinical wound isolate	https://www.atcc.org/
*S. aureus* ATCC 29213 Δ*coa*	*S. aureus* 29,213 *coa* deletion mutant	This study
*S. aureus* ATCC 29213 Δ*vwbp*	*S. aureus* 29,213 *vwbp* deletion mutant	[24]
*S. aureus* ATCC 29213 *ΔcoaΔvwbp*	*S. aureus* 29,213 *coa* and *vwbp* deletion mutant	[24]
*S. aureus* ATCC 29213 *coa:snap vwbp:clip*	*S. aureus* 29,213 producing both Coa:SNAP and vWbp:CLIP fusion proteins	This study
*S. aureus* ATCC 29213 *coa:msfgfp*	*S. aureus* 29,213 producing Coa:msfGFP	[24]
*Escherichia coli* IM08B	Methylates DNA to mimic methylation pattern of *S. aureus.* DNA cytosine methyltransferase deficient (Δ*dcm*) with added *S. aureus hsdMS* genes to methylate adenine residues	[28]
*Staphylococcus xylosus* C2a	Type strain carrying plasmid pSB2019	A gift from Prof. Friedrich Götz, Universität Tübingen

**Plasmid**	**Description**	**Reference**

pSB2019	Gram positive shuttle vector, constitutive *gfp3* expression, ampicillin and chloramphenicol resistance	[29]
pUC57-*CLIP*	*E. coli* vector carrying *CLIP*. Ampicillin resistance	Genscript
pUC57-*SNAP*	*E. coli* vector carrying *SNAP*. Ampicillin resistance	Genscript
pIMAY	*E. coli*/*Staphylococci* temperature sensitive vector for allelic exchange. Chloramphenicol resistance. Inducible *secY* antisense. pIMAY was a gift from Tim Foster (Addgene plasmid # 68,939; http://n2t.net/addgene:68939; RRID:Addgene_68,939)	[30]

**Primer**	**Sequence (5’ – 3′) and description**	**Reference**

v1F	atcaataaagtatacaatggcaaaTCAGGTGGTGGAGGAGATAA Forward primer to amplify CLIP sequence from pUC57-*CLIP*	This study
v1R	tttgcagccatgcattaatattaaccTAAACCTGGTTTACCTAAACG Reverse primer to amplify CLIP sequence from pUC57-*CLIP*	This study
v2F	tcactaaagggaacaaaagctgggtacCGTCAAACTCAGCAACAA Forward primer to amplify upstream of *vwbp* from *S. aureus* 29,213	This study
v2R	ttatctcctccaccacctgaTTTGCCATTGTATACTTTATTGAT Reverse primer to amplify upstream of *vwbp* from *S. aureus* 29,213	This study
v3F	cgtttaggtaaaccaggtttaggtTAATATTAATGCATGGCTGCAAA Forward primer to amplify downstream of *vwbp* from *S. aureus* 29,213	This study
v3R	gataccgtcgacctcgagggggggcccgCAAATAGCGTGCTCATAGTTAAA Reverse primer to amplify downstream of *vwbp* from *S. aureus* 29,213	This study
vOutF	AAAATCTAAAAATGAGTCTGTGGTT Forward primer for screening vWbp:CLIP integration	This study
vOutR	TTACTAACATTTACTTTTGGCGAAT Reverse primer for screening vWbp:CLIP integration	This study
c1F	atgggcctagagtaacaaaaTCAGGTGGTGGAGGA Forward primer to amplify SNAP sequence from pUC57-*SNAP*	This study
c1R	tgtctttggatagagttataaatttaACCTAAACCTGGTTTACCTAAA Reverse primer to amplify SNAP sequence from pUC57-*SNAP*	This study
c2F	cctcactaaagggaacaaaagctgggtacGCCAAGTGAAACAAACGCAT Foward primer to amplify upstream of *coa* from *S. aureus* 29,213	This study
c2R	ttatctcctccaccacctgaTTTTGTTACTCTAGGCCCATATGTC Reverse primer to amplify upstream of *coa* from *S. aureus* 29,213	This study
c3F	taggtaaaccaggtttaggtTAAATTTATAACTCTATCCAAAGACATACAGTCA Forward primer to amplify downstream of *coa* from *S. aureus* 29,213	This study
c3R	atcaagcttatcgataccgtcgacctcgagggggggcccgTTTTAAATTTTATGAATCGAAGCCCTTTG Reverse primer to amplify downstream of *coa* from *S. aureus* 29,213	This study
cOutF	GTGAAATATAGAGATGCTGGTACA Forward primer for screening Coa:SNAP integration	This study
cOutR	TGAAGTAGGCTGAAGTTGAAGC Reverse primer for screening Coa:SNAP integration	This study
IM151	TACATGTCAAGAATAAACTGCCAAAGC Anneals to pIMAY multiple cloning site	[30]
IM152	AATACCTGTGACGGAAGATCACTTCG Anneals to pIMAY multiple cloning site	[30]
coa A	GGGGGTCGACGTGCGCAGCTAAAATATCGCG *coa* deletion mutant	[24]
coa B	CCTCCAAAATGTAATTGCCCAATC *coa* deletion mutant	[24]
coa C	GATTGGGCAATTACATTTTGGAGGTCTATCCAAAGACATACAGTCAA *coa* deletion mutant	[24]
coa D	GGGGAGCTCGCGGGTTGAAGCAATTTCGTTT *coa* deletion mutant	[24]

For confocal microscopy experiments, bacteria were stained with the nucleic acid stain SYTO 41 (S11352, Life Technologies) when required, and SNAP and CLIP tags were stained with either SNAP-Surface Alexa Fluor 647 (S9136S, New England Biolabs) or CLIP-Surface 547 (S9233S, New England Biolabs), which are referred to as SNAP-647 and CLIP-547. Prior to staining, biofilms were blocked with 5 % bovine serum albumin (BSA, A9418, Sigma Aldrich) dissolved in 1 × phosphate buffered saline (PBS, 28,348, Thermo Fisher Scientific). In order to visualise fibrin, fluorescent fibrinogen conjugated to either Alexa-Fluor 647 (F35200, Thermo Fisher Scientific) or Alexa-Fluor 488 (F13191, Thermo Fisher Scientific) was added to biofilm growth medium, which became incorporated into the fibrin matrix, which are referred to as Fg-647 and Fg-488.

### 2.2 Construction of *S. aureus* mutants lacking coagulases

Deletion mutants of *S. aureus* 29,213 that lack Coa, vWbp, or both, were produced as described in Ref. [24]. Briefly, in-frame single deletions of the *coa* and *vwbp* genes were achieved through splicing by overlap extension PCR according to Monk and colleagues [30] and performed as described in detail in Wassmann et al., 2022 [31]. The double mutant was created by introducing the pIMAYΔ*coa* plasmid into the Δ*vwbp* mutant and deleting the *coa* gene in the Δ*vwbp* mutant. Coagulation assays of whole human blood were used to verify that Coa and vWbp could trigger coagulation alone without the presence of the other coagulase and are presented in Supplementary S1.

### 2.3 Construction of *S. aureus* mutants with genomically encoded fusion proteins

Mutants of *S. aureus* with genomically integrated *C*-terminal fusions of *coa:snap* and *vwbp:clip* were produced in the *S. aureus* 29,213 wildtype as described previously [24] (Table 1). After cloning, the correct genetic sequences were confirmed via PCR and Sanger sequencing using primer pairs cOutF/cOutR and vOutF/vOutR (Table 1), and the correct phenotype of the mutated strain was verified by comparison to the parental strain. The phenotype was assessed using coagulation assays and CLSM imaging. Data confirming that fusion to SNAP and CLIP did not inhibit the biological function of Coa and vWbp are presented in Supplementary S2.

### 2.4 Coagulation tests to assess coa and vWbp localisation in cell cultures

We investigated if Coa and vWbp were localised on the bacterial cell surface or secreted to the supernatant by testing the coagulation ability of cell culture supernatants or cells separated from supernatant. Overnight cultures of *S. aureus* 29,213 Δ*coa*, *S. aureus* 29,213 Δ*vwbp*, and *S. aureus* 29,213 Δ*coa*Δ*vwbp* were diluted 100 × in BHI supplemented with 5 % serum and grown to exponential phase (OD_600_ 0.3–0.4). Some of each culture was kept aside to use as a control sample in the coagulation tests, and the rest of the cells pelleted by centrifugation at 4000×*g* for 10 min. The supernatant was removed and filtered through a 0.2 mm polyether sulfone membrane filter (83.1826.001, Sarstedt) to remove remaining cells (“supernatant” sample). The pelleted cells were resuspended in BHI and some kept aside (“pelleted” sample). The remaining resuspension was washed twice in BHI by centrifugation and resuspension (“pelleted and washed” sample). 100 μl cells (control sample, pelleted, or pelleted and washed) or 143 μl supernatant were inoculated in sterile Hungate tubes with 1 ml 1:6 plasma in 0.85 % NaCl with 10 μg/ml *Cm*, and incubated overnight at 37 °C without shaking. Coagulation was observed by visual inspection. *Cm* was added to inhibit the production of new Coa or vWbp during the incubation with plasma, and to ensure that any coagulation seen was caused by Coa or vWbp produced before the initiation of the coagulation assay.

### 2.5 Transforming *S. aureus* 29,213 strains for *gfp* expression

Plasmid pSB2019 [29] was transformed using a modified version of the protocol in Ref. [32] via electroporation into *S. aureus* 29,213, *S. aureus* 29,213 Δ*coa*, *S. aureus* 29,213 Δ*vwbp*, and *S. aureus* 29,213 Δ*coa*Δ*vwbp* so cells would constitutively express GFP. pSB2019 was extracted from *Staphylococcus xylosus* C2a using the GeneJET Plasmid Miniprep Kit (K0502, Thermo Fisher Scientific). Electrocompetent *S. aureus* cells were prepared by first diluting an overnight culture to OD_600_ 0.5 and incubating until it reached OD_600_ 0.6.50 ml of cells were harvested by centrifugation at 4000×*g* at 4 °C and washed 3 × in 50 ml ice-cold Milli-Q water. Cells were then harvested and resuspended in 50 ml ice-cold 0.5 M sucrose (S7903, Sigma-Aldrich), and again in 5 ml, 2 ml, and finally 0.25 ml sucrose. 50 μl of electrocompetent cells were then incubated on ice with up to 5 μg pSB2019 for 10 min, then transferred to a chilled 1 mm electroporation cuvette and electroporated at 2.1 kV, 25 μF, and 200–300 Ω using the ECM 360 BTX (Harvard Apparatus). The resistance was varied to get a time constant τ > 4 ms. Immediately after electroporation, 1 ml preheated BHI with 0.5 M sucrose was added, after which the cells were incubated at 37 °C with shaking at 100–150 rpm for 2 h. Then cells were plated onto BHI plates containing *Cm* and incubated overnight. Colonies were screened for fluorescence by confocal laser-scanning microscopy (LSM700, Zeiss) using 488 nm excitation and 500–750 nm emission.

### 2.6 Biofilm preparation for fluorescence microscopy

Microwells (μ-Slide 8 Well, 80,821, IBIDI) were incubated with 180 μl mBHI supplemented with 50 % plasma and 0.004–0.4 μg/ml Fg-647 or Fg-488, if required, for 30 min at 37 °C to create a preconditioning layer. Overnight cultures of *S. aureus* were adjusted to OD_600_ 1–2, depending on the experiment, and 20 μl was added to the 180 μl preconditioning medium already in the microwell to reach a final OD_600_ 0.1–0.2. Biofilms were either imaged straight away (time-resolved CLSM), or incubated at 37 °C for 30 min to allow for early biofilm formation prior to imaging (static CLSM and HILO microscopy).

The inoculation procedure for static CLSM experiments with *S. aureus* expressing *coa:snap* and *vwbp:clip* was modified such that a single layer of surface-associated bacteria were used for inoculation in order to reduce background fluorescence from thick biofilms. After the initial incubation with preconditioning medium, the medium was removed and the wells were washed with 200 μl BHI. 100 μl bacteria adjusted to OD_600_ 10 were then added and incubated at 37 °C for 30 min. The non-attached bacteria were then removed and the wells were washed again with 200 μl BHI. 100 μl BHI supplemented with 50 % plasma and 0.4 μg/ml Fg-488 (if required) was finally added and incubated at 37 °C for 30 min.

### 2.7 Time-resolved CLSM of growing biofilms

Early biofilms of GFP-producing *S. aureus* 29,213, *S. aureus* 29,213 Δ*coa*, and *S. aureus* 29,213 Δ*vwbp* were grown and visualised using time-lapse CLSM with a microscope stage top incubator (H301, Okolab). Microwells were prepared and inoculated with OD_600_ 0.1 bacteria as described above in medium containing 0.4 μg/ml Fg-647. After allowing the cells to settle on the bottom of the well, they were located in brightfield, and time-lapse fluorescence imaging was started 10 min after inoculation. Z-stacks were obtained automatically every 10 min for a total run time of 160 min, and the setup autofocussed between each reading. A 10 mW 488 nm wavelength and 5 mW 639 nm wavelength laser were used for excitation operating at 2 % and 3 % power respectively, and a Plan-Apochromat 63x/1.40 oil immersion objective was used for imaging. The master gain was adjusted in a preliminary experiment to ensure that the fluorescent signal did not saturate the camera, and the same settings were subsequently used for each experiment. Z-stacks after 160 min of incubation were analysed qualitatively, and a series of slices from time-lapse Z-stacks were quantitatively analysed using a MATLAB code adapted from prior bespoke single-molecule particle tracking software [33], which can be downloaded from https://github.com/awollman/single-molecule-tools. The experiments were performed in triplicate.

### 2.8 Computational analysis of time-lapse data

The purpose of the analysis was to quantify how the mean intensity of fluorescence from fibrin in the vicinity of cells (the pseudocapsule) varied over the course of the time-resolved images. Time-lapse data was acquired as 16-bit greyscale images in two imaging channels, one for green emissions (GFP from bacteria) and one for red (Alexa-Fluor 647 from fibrinogen). The green channel was used to create a cell mask by calculating an intensity threshold using a locally adaptive intensity threshold with Bradley's method [34]. The sensitivity of the algorithm was decreased slightly to enable marginally more pixels to be encompassed in the foreground because the fibrin pseudocapsule localised to the surface of cells, not within them, and a morphological opening was used to remove small fluctuations in background that were misidentified as foreground objects. After the cell mask was generated, it was used to mask the red channel and calculate the mean fluorescence intensity from surface-associated fibrin within the cell mask at each time point. Data from three repeat images at each time point of each strain were analysed and the means and standard error in the means reported.

### 2.9 Photobleaching correction of time-lapse data

In order to adjust quantitative data to take into account photobleaching in the time-lapse images, correction factors were calculated for each time point. 70 μl Fg-647 (1.5 mg/ml) was incubated in the dark at room temperature for 1 h on a poly-lysine coated microscope slide (10149870, Thermo Scientific). It was washed 3 × with 100 μl PBS and imaged under identical conditions to the biofilm time-lapses. Data was processed using a bespoke MATLAB code that calculated the mean fluorescence intensity per frame and modelled the decay as an exponential function. The exponential decay constant λ was calculated and used to obtain correction factors for each frame using $e^{\lambda t}$ where t is the frame number. Data were collected from 3 repeat experiments. The mean correction factor and corresponding standard error in the mean for each time point for these data were calculated and used to correct the time-lapse analysis.

### 2.10 Static CLSM of Coa:SNAP and vWbp:CLIP fusion proteins in *S. aureus* biofilms

Biofilms of *S. aureus* 29,213 *coa:snap vwbp:clip*, and the parental strain were prepared as described above. After 30 min biofilm growth, biofilms were blocked with 100 μl blocking buffer (5 % BSA in 1 × PBS) for 30 min at room temperature. Afterwards, the blocking buffer was replaced with staining buffer (5 % BSA in 1 × PBS with 1 μM Syto 41 and 0.5 μM SNAP-647 or CLIP-547) and incubated for a further 30 min at 37 °C. The biofilms were then washed with 100 μl blocking buffer by incubating at room temperature for 1 h. The wash step was repeated once more and finally the biofilms were covered with 100 μl blocking buffer and imaged with CLSM. Biofilms were imaged with 5 mW 405 nm, 10 mW 488 nm, 10 mW 555 nm, and 5 mW 639 nm wavelength lasers operating at 2 %, 2 %, 2 %, and 3.5 % power respectively, using a Plan-Apochromat 63x/1.40 oil immersion objective lens, 6–21 μs pixel dwell time, and averaging over 16 line scans.

### 2.11 HILO microscopy of Coa:msfGFP in *S. aureus* biofilms

Biofilms of *S. aureus* producing either Coa:msfGFP or unmodified Coa were prepared in the same way as described above with an initial OD_600_ of 0.2 and an Fg-647 concentration of 4 ng/ml. A control sample of fluorescent beads (TetraSpeck Microspheres 0.2 μm, T7280, Invitrogen) was prepared by diluting beads 1/100 in 1 x PBS and pipetting 50 μl into a tunnel created between a plasma-cleaned #1.5 coverslip and a microscope slide adhered together with 3 M Scotch tape as described previously [35].

Samples were imaged with a bespoke single molecule fluorescence microscope with HILO illumination at an angle of 45° and with a laser beam profile of 25 μm. Biofilms were alternately excited at a 50 ms frame rate for 300 frames by 640 nm and 488 nm wavelength lasers (OBIS 640 nm LX 40 mW and OBIS 488 nm LX 50 mW) operating at 1 mW and 40 mW, and fluorescence emissions were split with a dual channel simultaneous imaging system (DV2, Photometrics) according to wavelength into separate green and red channels. For each field of view, a second image was recorded of the sample with brightfield microscopy. Fluorescent bead samples were alternately excited at a 50 ms frame rate for 10 frames by 640 nm and 488 nm wavelength lasers operating at 1 mW.

### 2.12 HILO distribution analysis

#### 2.12.1 Segmentation and 2D distance map

Brightfield video sequences were averaged over 10 frames and background subtracted, using an empty scene, to generate clear images for segmentation. The cell boundaries were segmented into binary ROIs manually using the ImageJ [36] ellipse tool. The binarised cell boundaries were converted into a pixelwise in-plane Euclidean distance map using the ImageJ ‘Distance Map’ function. The distance values inside the cell ROIs were multiplied by −1 to distinguish intracellular and extracellular domains.

#### 2.12.2 Radial distributions of coa and fibrin

The alternately-excited frames in each HILO video sequence were deinterleaved and cropped to generate sequences corresponding to i) the Coa:msfGFP channel under 488 nm excitation only and ii) Fg-647 under 640 nm excitation only. Frames 1–3 and 130–150 in each channel were averaged separately to establish the respective fluorescent signals before and after complete photobleaching of the fluorophores of interest. The latter ‘after’ image, capturing the majority of the autofluorescent and out-of-focus light, was subtracted from the first ‘before’ image to extract the fluorescent component corresponding to either in focus Coa:msfGFP or Fg-647, alongside any residual background. The Fg-647 channel was registered laterally onto the GFP channel using an affine transform generated from images of fluorescent beads appearing in both channels. The intensity pixel values in the registered images were then tabulated alongside the distance values for each pixel. These intensities were divided by the characteristic single molecule intensity to generate numbers of molecules per pixel. This was divided by the estimated 400 nm optical depth of field and Avogadro's constant to yield volumetric concentrations in molar units. The concentration values were collated into 10 nm distance bins, smoothed by a 53 nm (pixel-width) Gaussian kernel, and reported as mean concentration ±95 % confidence interval for each distance bin.

#### 2.12.3 Single molecule characteristic intensity

10 nM solutions of monomeric GFP derived from competent *E. coli* [37] or free Alexa-Fluor 647 dye in PBS were pipetted between a plasma-cleaned #1.5 coverslip and slide adhered together with 3 M Scotch tape as described previously [35]. The slides were imaged using the same excitation and acquisition settings as *S. aureus* biofilms. Fluorescent foci and the Chung–Kennedy-filtered photobleaching steps in their intensities were identified from the images using ADEMScode [38]. The modal brightness was found to be 228 ± 39 (n = 996) and 152 ± 25 (n = 511) pixel grey values per label for individual Alexa-Fluor 647 and mGFP respectively, the latter consistent with intensity step sizes of mGFP inside other bacterial cells *in vivo* [39].

#### 2.12.4 CLSM of *S. aureus* grown with and without antibiotics

Overnight cultures of wildtype GFP-producing *S. aureus* 29,213 were and adjusted to OD_600_ 0.1 in either BHI or BHI supplemented with 10 μg/ml erythromycin. 200 μl of adjusted cultures were added to microwells and incubated for 160 min while simultaneously recording a time-lapse via time-resolved CLSM using the method described above, with the exception that only the 488 nm laser was used and only green emissions from GFP were collected.

## 3 Results

### 3.1 Coa and vWbp are loosely associated with the cell surface

To understand how Coa and vWbp might contribute differently to fibrin production in *S. aureus* biofilms, we first investigated whether these coagulases associate with the cell surface or if they are secreted to the supernatant. We separated cells from the supernatant by centrifugation and filtration and tested the coagulation ability of *S. aureus* mutants lacking either vWbp, Coa, or both (Table 2). We added *Cm* to the coagulation tests to inhibit protein synthesis and ensure that coagulation only occurred due to Coa or vWbp produced while cells grew in BHI with 5 % serum prior to the coagulation test. We selected the culture conditions based on a preliminary experiment, which revealed that the host factors in serum increased the production of Coa and vWbp (Supplementary S3).

**Table 2:** Tube coagulation tests for *S. aureus* mutant strains added to human plasma containing chloramphenicol. *S. aureus* was cultured in BHI with 5 % serum until the stationary growth phase before transferring to human plasma. The culture was either diluted directly in plasma, or cells were separated from the supernatant by centrifugation and filtration. In one experiment, cells were washed by two centrifugation and resuspension steps to remove loosely bound surface proteins.

	Coagulation (±)			
	Diluted bacterial culture	Pelleted bacteria	Pelleted and washed bacteria	Sterile-filtered culture supernatant
**Wildtype**	**+**	**+**	**+**	**+**
**Δ*vwbp***	**+**	**+**	**-**	**+**
**Δ*coa***	**+**	**+**	**-**	**+**
**Δ*coa*Δ*vwbp***	**-**	**-**	**-**	**-**

In samples containing either Coa or vWbp, coagulation occurred when adding both cells or supernatants to human plasma, suggesting that both proteins at least partly associate with the cell surface of *S. aureus*. To test how firm the association with the cell surface is, we washed the cells by multiple centrifugation and resuspension steps prior to the coagulation test. After this procedure, only the wildtype cells caused coagulation. Hence, we conclude that Coa and vWbp are only loosely associated with bacterial cells although the mechanism is not known. As expected, the double mutant did not cause coagulation at all, confirming that Coa or vWbp were responsible for coagulation in the other samples.

### 3.2 Coa is essential for pseudocapsule formation

In *S. aureus* biofilms, coagulation leads to fibrin located as a pseudocapsule around cell clusters and an extended network of fibrin fibers in the wider biofilm matrix [8]. Previous studies have suggested that Coa is primarily responsible for pseudocapsule formation, while vWbp promotes the formation of matrix-associated fibrin, because Coa associates to cell surfaces and presumably vWbp does not [8]. However, our investigation indicated that both coagulases associate with the cell surface. To explore their potentially different roles in *S. aureus* biofilms, we visualised fibrin in biofilms of *S. aureus* wildtype and mutants lacking Coa, vWbp, or both, after 160 min of incubation in media containing 50 % human plasma.

**Fig. 1:**
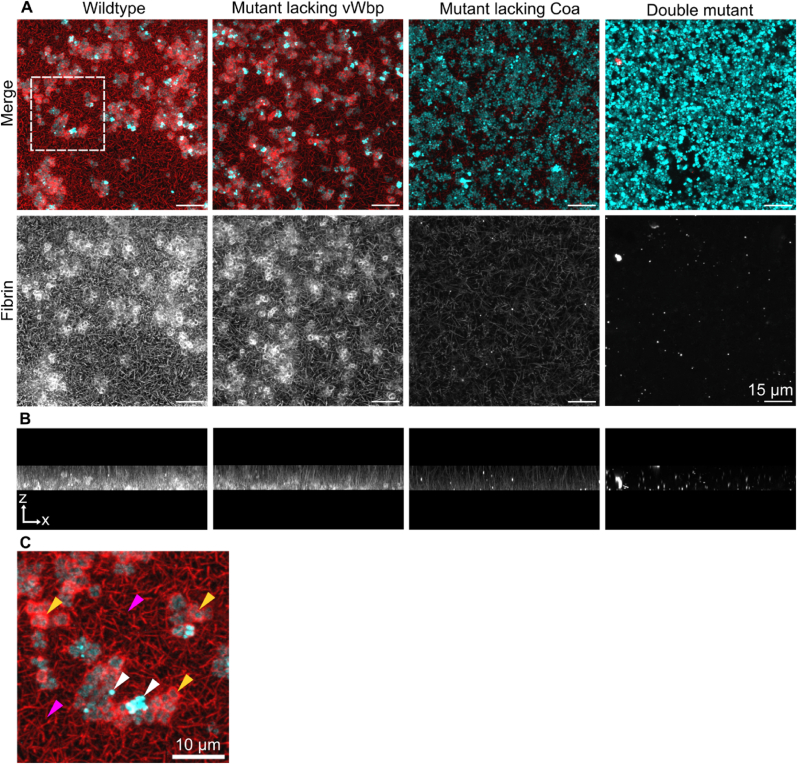
**Pseudocapsule formation requires Coa. a)** Maximum intensity Z projections of *S. aureus* biofilms formed after 160 min of incubation in 50 % human plasma. The white dotted square represents the location of a zoomed in image presented in c). **b)** Maximum-intensity projection of *S. aureus* wildtype and mutant biofilms rotated about the x-axis to display the x-z side-profile of fibrin fibers arranged perpendicular to the glass substrate. **c)** Zoomed in image of an *S. aureus* wildtype biofilm. Yellow arrows indicate pseudocapsules, magenta arrows indicate the extended fibrin network, and white arrows indicate bacteria that lack pseudocapsules. Bacteria are cyan and visualised by GFP expression and fibrin is red/white and visualised by addition of Alexa Flour-647 labelled fibrinogen to the biofilm growth medium. All images were acquired with the same settings.

Fibrin formed as pseudocapsules around cells and as a network of fibrin fibers extending between cell clusters in wildtype *S. aureus* biofilms (Fig. 1a). Fibrin fibers even extended from the glass surface (Fig. 1b), indicating nucleation of fibrin formation at the solid/liquid interface. Pseudocapsules formed around single cells and cell clusters (Fig. 1c, yellow arrows), but not all bacteria had a pseudocapsule. The capsule-free bacteria also had brighter fluorescence than the others (Fig. 1c, white arrows).

When vWbp was lacking, the biofilm phenotype looked similar to the wildtype, but when Coa was lacking, the cells did not form a pseudocapsule (Fig. 1a). As expected, no fibrin formed in the double mutant (Fig. 1a). This is in agreement with a previous study, which concluded that vWbp promotes the formation of an extended fibrin network but not a pseudocapsule [8]. In contrast to the previous study, our results indicate that Coa contributes to the formation of both the pseudocapsule and the extended fibrin network, as indicated by the strongly fluorescent fibrin network in the wildtype strains compared to the mutant lacking Coa. vWbp activates prothrombin more slowly than Coa [15], and having both coagulases might accelerate fibrin production in the entire biofilm. We observed that vWbp contributed relatively more to the coagulation of whole blood during a 48 h incubation than Coa (Supplementary S1), yet the higher fluorescence intensity from fibrin in the mutant lacking vWbp (Fig. 1a and b) indicates that Coa had a greater contribution than vWbp to coagulation in early biofilms grown for 160 min. These results highlight temporal differences in the activity of both coagulases and underlines the importance of both coagulases for full coagulation.

### 3.3 vWbp accelerates pseudocapsule formation

The kinetics of fibrin formation in biofilms can reveal more about the roles of Coa and vWbp. Coa is required to produce the pseudocapsule, and fibrin growth dynamics at the cell surface would indicate whether Coa and vWbp truly play individual roles in pseudocapsule formation, or whether there is a cooperative effect of their activity. We therefore quantified the formation of cell surface-associated fibrin during the initiation of biofilm formation using time-lapse confocal microscopy.

**Fig. 2:**
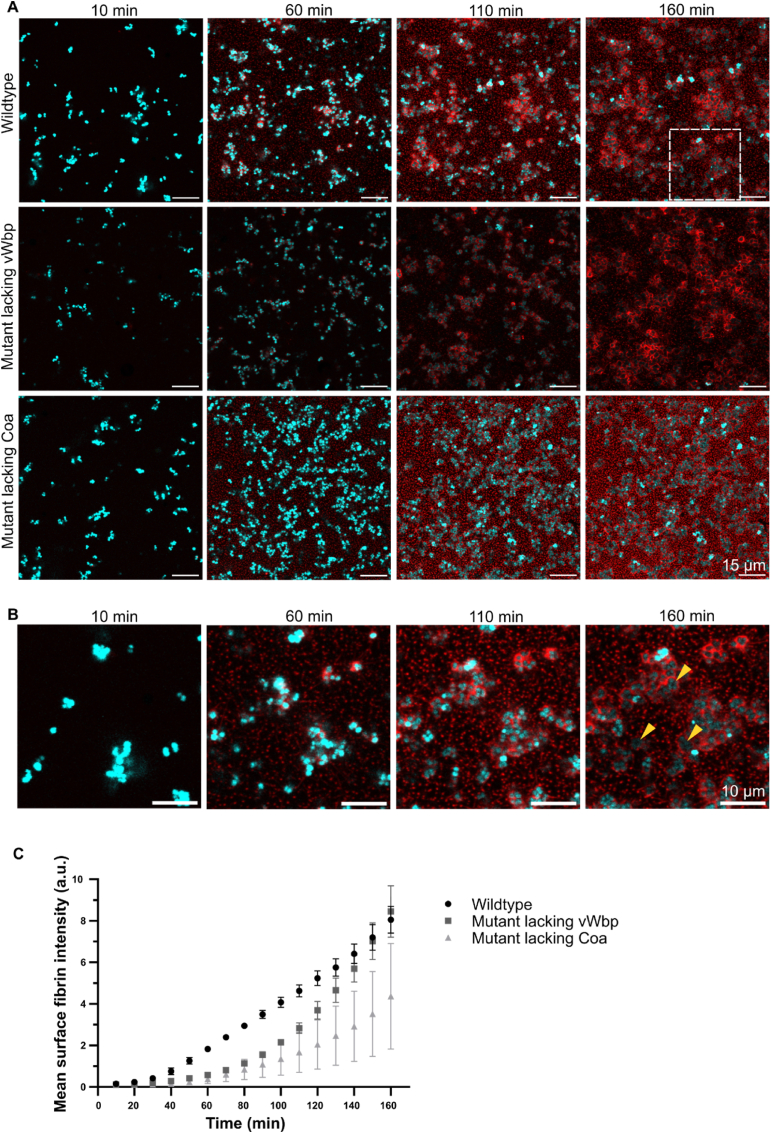
**vWbp accelerates pseudocapsule formation. a)** Time lapse microscopy of *S. aureus* biofilms growing in media supplemented with 50 % plasma. Z slice 5/30 is presented. Cyan = bacteria, red = fibrin. The white square indicates the area shown in b). **b)** Zoomed image of bacteria replicating inside shared pseudocapsules. Yellow arrows point to examples. **c)** The mean fluorescence intensity from surface-associated fibrin increased over time for the wildtype and bacteria lacking vWbp. Fluorescence intensity increased for the mutant lacking Coa but was not associated to the surface of bacteria and instead intersected with bacteria. 192–380 bacteria were included in the calculations at time = 10 min, which rose to 618–2092 at time = 160 min. 3 biological replicates of each strain were used. Error bars show the standard error of the mean.

Time-lapse imaging verified the end-point measurements from Fig. 1, showing distinct morphotypes of fibrin in biofilms formed by the different mutant strains (Fig. 2a, supplementary videos). It also revealed that cell clusters with a shared pseudocapsule originated from single cells that had formed a pseudocapsule before undergoing cell division (Fig. 2b, Supplementary videos). Aggregating within a shared pseudocapsule may protect bacteria from phagocytosis, which is impaired for aggregates larger than 5 μm in diameter [40].

The fluorescence intensity of surface-associated fibrin indicated an initial lag of approximately 30–50 min followed by a continuous increase (Fig. 2c). The shortest lag phase was in the wildtype strain, and the longest was in the mutant strain lacking Coa. The lag phase represents the time required for Coa and vWbp to be secreted and form an active complex with prothrombin and cleave fibrinopeptides A and B from fibrinogen, and for the fibrin monomers to assemble longitudinally to form protofibrils. The subsequent rise in fluorescence intensity occurs due to the aggregation of fibrin monomers laterally and longitudinally to form fibrin fibers. Formation of surface-associated fibrin in the mutant strain lacking vWbp was slower than the wildtype strain at the start of the time-lapse, but it eventually “caught up” as pseudocapsules were fully formed (Fig. 2a–c). In the mutant strain lacking Coa (where pseudocapsules were absent), the fluorescent signal from surface-associated fibrin originated from fibrin fibers in the extended network that happened to intercept with *S. aureus* cells, and the signal was therefore lower and much more variable.

If Coa was solely responsible for pseuodocapsule formation, we would expect that the change in signal intensity in surface-associated fibrin would be similar in the wildtype and the mutant lacking vWbp. However, there was a delay in the increase of fluorescence from surface-associated fibrin in the mutant strain (Fig. 2c). This result suggests that the two coagulases work cooperatively to accelerate pseudocapsule formation. Hence, vWbp cannot form a pseudocapsule *per se*, but it accelerates pseudocapsule formation by Coa.

### 3.4 Coa and vWbp both locate to cell surfaces in *S. aureus* biofilms

Previous studies showed that Coa localises strongly within the fibrin pseudocapsule and accumulates at the periphery of *S. aureus* abscess lesions, while vWbp is distributed throughout abscesses and also accumulates at the periphery [20]. Coa has also been visualised in the fibrin pseudocapsule with immunolabelling [8] and fusion to msfGFP [24]. To directly address the localisation of Coa and vWbp with respect to cells and fibrin in biofilms, we developed fusion proteins Coa:SNAP and vWbp:CLIP in *S. aureus* and visualised them by CLSM. We omitted fluorescent fibrin from experiments with vWbp:CLIP due to overlap in emission spectra between the CLIP tag and fluorescent fibrinogen. We also visualised a fusion of Coa and msfGFP [24] and fluorescent fibrin using HILO microscopy, which provided improved imaging resolution compared to CLSM and allowed us to quantify average concentrations of fluorescent Coa and fibrin in the biofilm matrix relative to distance from cells. We used the Coa:msfGFP fusion protein instead of Coa:SNAP to avoid issues of fluorescent background from non-specific binding of the SNAP tag substrate, which might interfere with sensitive imaging methods.

**Fig. 3:**
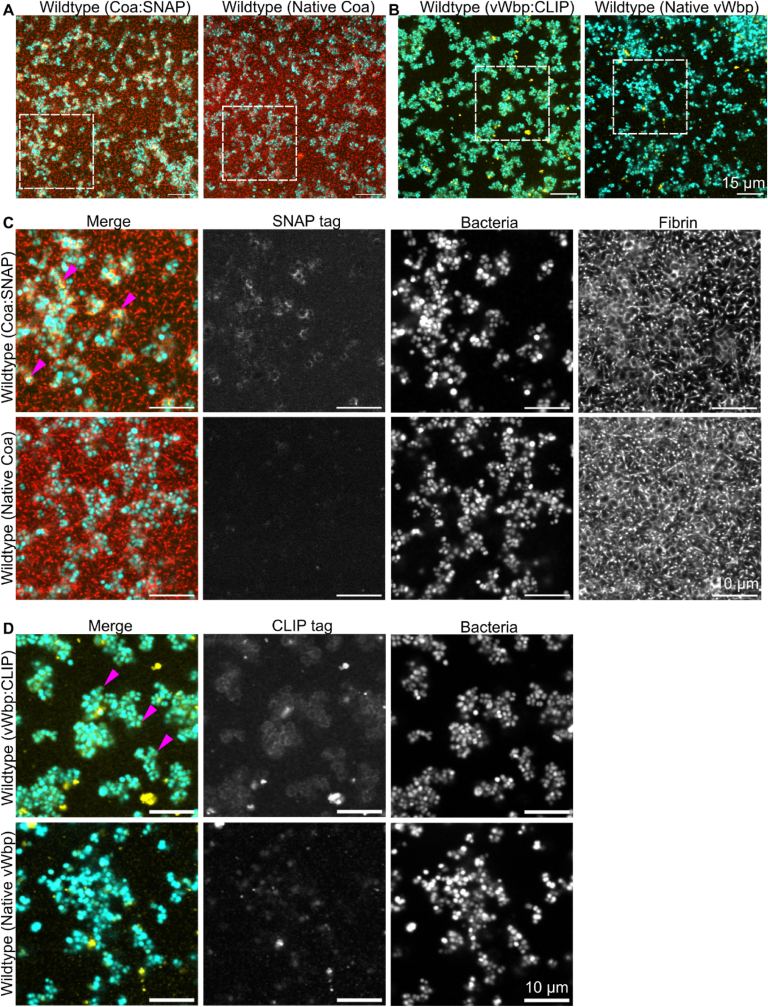
**Coa and vWbp locate to cell surfaces.** 2 h *S. aureus* biofilms grown in media supplemented with 50 % human plasma stained for either **a)** Coa:SNAP or **b)** vWbp:CLIP. Dotted white boxes indicate areas that are magnified in c) and d). **c)** and **d)** are magnified images of SNAP- and CLIP-stained biofilms. Both Coa:SNAP and vWbp:CLIP localised to bacterial surfaces. Magenta arrows indicate examples. Fluorescent fibrin was omitted when imaging vWbp:CLIP due to cross-talk arising from overlap in the emission spectra of the CLIP tag substrate and fluorescent fibrinogen. Bacteria are cyan and visualised by SYTO 41, fibrin is red and visualised by addition of Alexa Flour-488 labelled fibrinogen to the biofilm growth medium, and Coa:SNAP and vWbp:CLIP are yellow and visualised by SNAP-647 and CLIP-547.

Both Coa:SNAP and vWbp:CLIP localised to the surface of bacteria, where we hypothesised that they would catalyse fibrin production to produce the pseudocapsule (Fig. 3a–d). Zoom-ins of the images in Fig. 3a and b (shown in Fig. 3c and d) reveal that there is a weak surface-associated and ring-shaped fluorescence signal from Coa:SNAP and vWbp:CLIP (top). The fluorescent signal appeared relatively dim due to the difficulty of staining and imaging through the complex biofilm matrix. Fluorescence in the parental strain biofilms shows a weak fluorescent signal that originates from autofluorescence, non-specific binding, and aggregation of the SNAP and CLIP tag substrates. However, the absence of ring-shaped fluorescence at the surface of bacteria in the parental strains confirms that the fluorescence seen in Fig. 3c and d (top rows) originated from surface-associated Coa:SNAP and vWbp:CLIP.We could not detect fluorescence from any fusion proteins that were not associated with bacterial cells, but this may be due to the protein concentration being too low to be detected using standard CLSM.

**Fig. 4:**
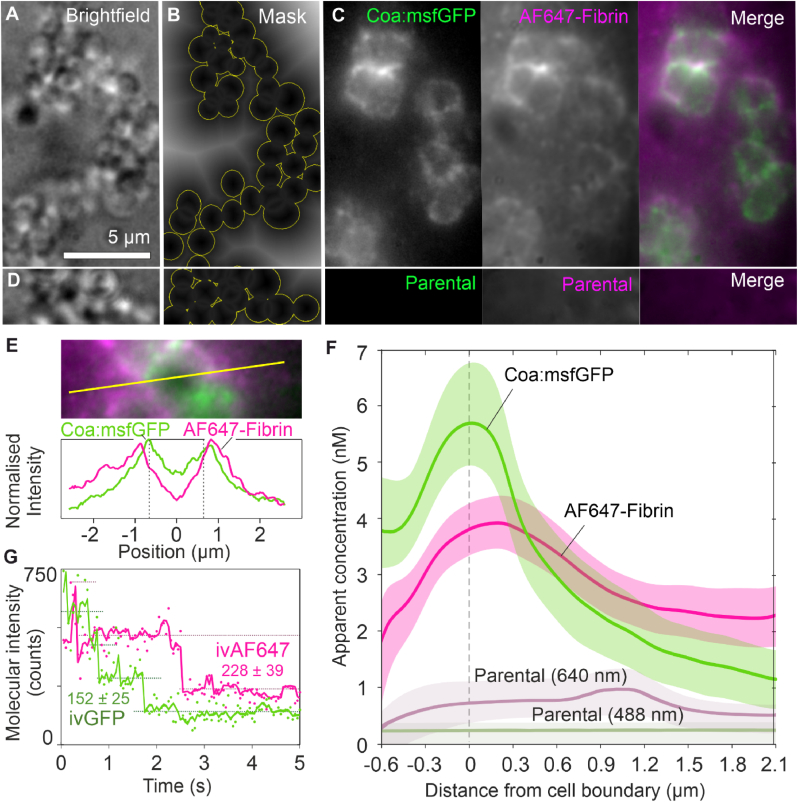
**Coa is highly concentrated at cell surfaces.** Coa:msfGFP and fibrin are concentrated at the cell boundary in *S. aureus* biofilms consistent with extracellular synthesis of fibrin. **a)** Brightfield illumination enables estimation of **b)** proximity to cell boundaries using a distance map transform. **c)** HILO imaging of the same cells yields the diffraction-limited fluorescent intensity distributions of Coa:msfGFP and Alexa Fluor-647 (AF647)-labelled fibrin. **d)** Parental cells without labelling on proteins or fibrin. **e)** Two-colour cross-sectional fluorescent intensity profile of an individual cell. **f)** Apparent average concentration profiles relative to the cell boundaries (mean ± 95 % CI) derived by comparing background-subtracted fluorescence images with cellular distance maps (n > 200 cells in >10 fields of view per condition). The true profiles are blurred by the diffraction limit (circa. 200 nm) and include out-of-focus contributions, such that fibrin is apparent at a non-zero concentration inside the cells. **g)** Consistent photobleaching step intensities of foci containing overlapping molecules of mGFP or AF647 *in vitro* under the same imaging conditions, facilitating calibration of fluorescent intensities to approximate concentrations of Coa:msfGFP and fibrin-associated AF647 fluorophores *in vivo*.

While CLSM is usually considered the gold standard for imaging biofilms due to its strong ability to optically section samples and reject out-of-plane background, CLSM produced a very low signal to noise ratio when imaging Coa:SNAP inside biofilms, making it unsuitable for further quantification (Fig. 3C). Therefore, we applied HILO microscopy of a Coa:msfGFP fusion [24] to gain higher quality images, a method which has not been extensively explored for imaging biofilms. HILO increases the signal to noise ratio compared to epifluorescence microscopy by increasing fluorescence and decreasing background and has been applied for single-molecule applications [41]. Using a fusion protein with msfGFP rather than the SNAP tag allowed us to avoid background noise that arose from non-specific binding of the fluorescent SNAP tag substate. By combining HILO illumination with a high excitation intensity and millisecond sampling rate, we obtained higher quality images that enabled us to perform quantitative analyses on the images and determine the average concentrations of Coa and fluorescent fibrin with respect to distance from cells in biofilms (Fig. 4). Firstly, HILO microscopy of Coa:msfGFP confirmed that Coa localises to cell surfaces, and subsequent quantification of HILO microscopy data revealed that Coa and fibrin are both highly concentrated at cell surfaces. We used brightfield images of *S. aureus* biofilms (Fig. 4a) to create cell masks to locate cell boundaries (Fig. 4b) and analyse Coa:msfGFP (Fig. 4c) relative to the cell boundary. The parental strain of *S. aureus* was used as a negative control, and confirmed that fluorescence could be ascribed to Coa:msfGFP and fluorescent fibrin, and not autofluorescence (Fig. 4d). Fluorescence intensity from Coa:msfGFP and fluorescent fibrin was larger at cell boundaries and decreased with distance from cells (Fig. 4e and f). Quantifying foci from single molecules of mGFP and Alexa Fluor-647 that were imaged under the same conditions as biofilms (Fig. 4g) allowed the conversion of fluorescence intensities to estimates of apparent concentrations, which demonstrates that Coa is highly concentrated at the cell surface. Together, these data are consistent with our hypothesis that Coa associates to cells and facilitate highly localised fibrin production at cell surfaces.

### 3.5 Only actively growing bacteria form a pseudocapsule

The time-lapse imaging of fibrin production made it apparent that some bacteria did not form a pseudocapsule, and that the fluorescence from GFP in these bacteria remained bright during the 160-min incubation, while the signal from other bacteria in the biofilm became dim (Fig. 1, Fig. 2b). Approximately 2 % of the total bacteria had bright fluorescence by the end of the 160 min incubation. In a further experiment, GFP fluorescence intensity in *S. aureus* appeared bright in non-dividing cells from a stationary-phase culture, and dim in exponential-phase cultures (data not shown) where the rate of GFP production cannot keep up with the rate of cell division [42]. We therefore hypothesised that the brightly fluorescent cells were inactive cells that were not dividing and not secreting Coa to enable pseudocapsule formation.

**Fig. 5:**
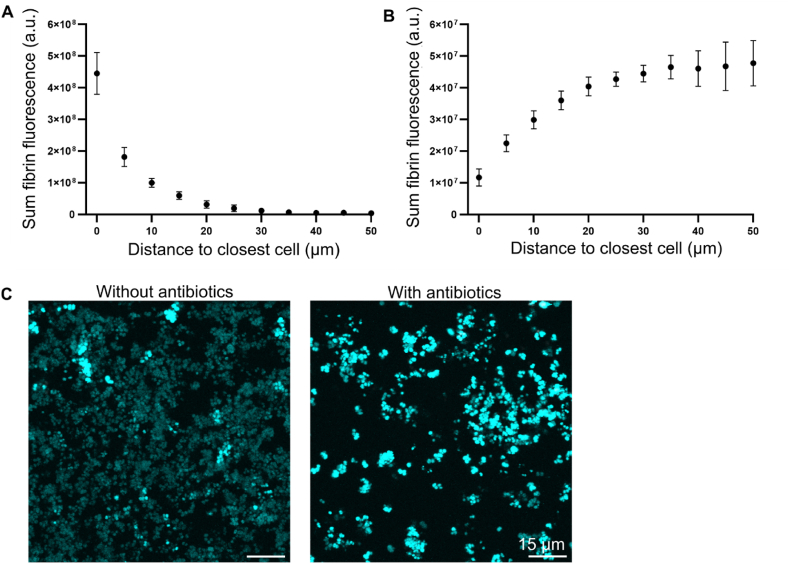
**Non-dividing cells have bright GFP fluorescence. a)** The sum of fluorescence intensity from fibrin plotted against distance from cells when including all cells in the image and **b)** when including only the bright cells. Final frames from time lapses were segmented to create masks containing either all the cells, or the bright cells only. A distance transform was applied to the cell mask, and then distances were binned into bins of width 5 μm and the sum of the fluorescence intensities from pixels falling into those distance ranges calculated. Calculations were performed on the final frame of the wildtype timelapses, with 618–1100 bacteria per calculation. 3 biological replicates were used. Error bars show standard error of the mean. **c)** Maximum intensity Z-projections of the final frame (160 min) of a time lapse of *S. aureus* incubating in either BHI or BHI supplemented with 10 μg/ml erythromycin. Plasma was not added to the growth medium during this experiment and therefore the biofilms presented do not contain fibrin. In the presence of erythromycin, bacteria remained bright during the time lapse, whilst most of them became dim in the absence of erythromycin.

To quantify the absence of pseudocapsule formation in this sub-population of cells in the biofilm, we segmented the cells in microscopy images based on their fluorescence intensity from GFP and then quantified the surface-associated fibrin in the bright cells. We analysed the final image from each time-lapse of the wildtype strain and compared to the fluorescence intensity from surface-associated fibrin for all of the cells in that frame. Plotting the fibrin signal as a function of distance to the nearest cell showed that the fluorescence intensity was highest at the surface of cells and decreased with distance from the cells (Fig. 5a). When analysing the bright cells only, the opposite was true (Fig. 5b). This confirms that the bright cells are a distinct sub-population that does not form a pseudocapsule.

To assess whether the variation in GFP fluorescence intensity reflected differences in the growth rates, we imaged *S. aureus* growing in either BHI or BHI supplemented with erythromycin, which inhibits protein synthesis and prevents cells from dividing. As expected, *S. aureus* grown in BHI split into the bright and dim subpopulations, while those incubated with erythromycin remained brightly fluorescent (Fig. 5c). Collectively, the results show that actively growing cells are responsible for pseudocapsule and cell cluster formation.

## 4 Discussion

We have shown that the two coagulases produced by *S. aureus* perform different but cooperative roles in the production of fibrin in *S. aureus* biofilms. Both coagulases associate non-covalently with the bacterial cell surface (Table 2, Fig. 3), and by quantifying high resolution HILO images using single-molecule techniques, we estimated the concentration of Coa molecules in the *S. aureus* biofilm matrix with respect to cell positions (Fig. 4). While Coa is primarily responsible for forming a pseudocapsule around single cells or cell clusters (Fig. 1a), we show for the first time that vWbp accelerates pseudocapsule formation (Fig. 2c). Both coagulases contribute to the formation of a wider fibrin network that connects cell aggregates and generates an extracellular matrix around the biofilm at large (Fig. 1a).

These findings agree with our previous study, where we visualised Coa at the surface of *S. aureus* cells in biofilms using a fusion protein of Coa with msfGFP and CLSM [24]. Our findings also partially agree with the study from Guggenberger et al. (2012), who showed that Coa is responsible for formation of the pseudocapsule and not matrix-associated fibrin, whilst vWbp is responsible for forming matrix-associated fibrin and only a partial pseudocapsule [8]. Our study is also in agreement with Cheng et al. (2010), who detected Coa within the pseudocapsule and in the periphery of *S. aureus* abscess lesions, while vWbp was distributed throughout the infection as well as at the periphery [20]. Perhaps the partial pseudocapsule in Ref. [8] is formed by fibrin in the wider biofilm that intercepts with *S. aureus*, rather than resulting from highly localised fibrin production caused by surface-associated Coa.

Formation of the pseudocapsule is important *in vivo* because it acts as a barrier against host immune cells and protects bacteria from phagocytosis [8,43]. Pseudocapsule formation prevents opsonisation: the secreted protein Efb binds to complement factor C3b on the surface of opsonised bacteria and attracts fibrinogen, which inhibits the detection of C3b and prevents phagocytosis by neutrophils [44]. The acceleration of pseudocapsule formation by vWbp may be critical for survival in the host, as it enables faster activation of the pathogen's defence mechanism. While the pseudocapsule itself is a protective shield, it also appears to play a role in forming bacterial cell clusters, which is another defence mechanism by the pathogen. After a few cell divisions, clusters become sufficiently large to exceed the size limit of what can be engulfed by neutrophils and macrophages by phagocytosis. Our time-lapse microscopy revealed that cell clusters were formed by clonal expansion from a single cell. The pseudocapsule constrained new cells arising from cell division, leading to bacterial growth that resulted in cell clusters growing within a shared pseudocapsule (Fig. 2b).

Although vWbp does not initiate pseudocapsule formation, we observed a weak ring-shaped fluorescence from vWbp in the pseudocapsule (Fig. 3d). vWbp can bind to the surface of *S. aureus* via the MSCRAMM ClfA, where it forms a complex with von Willebrand factor that associates bacteria to endothelial cells [45]. We assume that vWbp has not formed an activated complex with prothrombin to trigger coagulation in this case; otherwise, we would observe pseudocapsule formation in the absence of Coa. We therefore speculate that the activated complex of prothrombin with vWbp associates to the pseudocapsule after its formation has already been initiated by Coa. Coa only binds to fibrinogen and prothrombin [17,18] and is, presumably, recruited to the bacterial surface using fibrinogen as a bridging molecule where it then produces fibrin highly localised to the cell surface. vWbp on the other hand has ligands that bind fibronectin and von Willebrand factor, and can bind to and activate factor XIII, which in turn crosslinks fibrin fibers [13,19]. We therefore suggest that the vWbp-prothrombin complex is recruited to the pseudocapsule via interaction with fibronectin or von Willebrand factor which may be present in the pseudocapsule, or via factor XIII crosslinking of fibrin in the pseudocapsule. It is also possible that interaction with these other host components could limit fibrin production as the biofilm matures.

We also observed fibrin fibers extending perpendicularly from the glass substrate in samples with either Coa or vWbp, demonstrating a capacity to initiate fibrin formation away from the bacteria (Fig. 1b). *In vivo*, fibrin formation that does not form at the bacterial cell surface could provide an opportunity for bacterial attachment to host tissue, as Staphylococci are equipped with multiple cell wall-anchored fibrin-binding proteins. *S. aureus*-derived fibrin facilitates bacterial adhesion to multiple infection sites, including catheters [22], heart valves [46], and vascular cells [47]. Such bacteria-derived fibrin deposition may thus affect host colonisation in a broader sense by providing new sites for *S. aureus* colonisation and cause hematogenous spread of the infection. Indeed, a new study points to vWbp as critical for *S. aureus*’ ability to move from the blood stream to the joints and cause septic arthritis [48]. This study highlights the need to further investigate the differential role of Coa and vWbp in different types of infections.

Cell-to-cell variation in GFP fluorescence intensity revealed a sub-population of brightly fluorescent cells that did not form a pseudocapsule and presumably did not contribute to biofilm formation at all. We confirmed that the bright GFP fluorescence was indicative of slow or arrested cell growth (Fig. 5). Roostalu et al. (2008) [42] also identified a subpopulation of non-dividing *E. coli* which could also be distinguished by their bright GFP signal when using *gfpmut2* expressed from the plasmid pMSLuxR after this population was highly tolerant to antibiotics. The authors suggested that non-growing antibiotic-tolerant cells were persister cells, which are responsible for chronic and recurring infections due to their antibiotic tolerance [42]. The non-growing cells in our study could also be cells that tolerate antibiotics that require active cell growth or a high metabolic rate to take effect. These cells might be part of a bet-hedging strategy [49], whereby they do not contribute to the energy-requiring pseudocapsule formation yet become essential for the population's survival later on during antibiotic therapy. While these cells do not contribute to biofilm formation, they may become essential for re-growth of the bacterial population if the other, non-dormant cells, are eradicated by antibiotics.

Coagulation is a critical capability for the survival and proliferation of *S. aureus* during infections. This mechanism is therefore a potential target for preventing *S. aureus* infections through vaccination [50], or development of new therapies that attenuate *S. aureus* virulence. A recent study identified a small molecule which inhibits Coa, resulting in improved therapeutic outcome from standard antibiotics against a lung infection [51], and there are a number of studies that investigate other molecules that inhibit coagulation either by inhibiting Coa or the active complex with prothrombin [21,[52], [53], [54]]. Our study adds more detail to the understanding of Coa and vWbp's location and function during the early stages of biofilm formation, and these insights are valuable for understanding how therapies directed at *S. aureus* coagulation will affect its phenotype and survival.

## 5 Conclusion

*S. aureus* secretes Coa and vWbp to build a matrix of fibrin fibers consisting of a pseudocapsule and an extended network of fibrin fibers. Host factors increase the production of Coa and vWbp and loosely associate them to cell surfaces. Coa is necessary for forming a surface-associated fibrin pseudocapsule, whilst vWbp accelerates its formation. Coa and vWbp both associate to cell surfaces to produce the pseudocapsule, and both Coa and vWbp appear to form fibrin in the wider biofilm too. Hence, their roles are slightly different but overlap to a degree. Coa appears to form both fibrin structures on its own, while vWbp forms fibrin in the wider biofilm and accelerates pseudocapsule formation in the presence of Coa. vWbp binds a wider array of host proteins than Coa, so its role could be to reinforce the existing fibrin network and mediate attachment to host tissue. *S. aureus* biofilms also contain small sub-populations of cells that do not produce a pseudocapsule and appear to be non-dividing, which may represent a bet-hedging strategy used to persist antibiotic therapy.

## CRediT authorship contribution statement

**Dominique C.S. Evans:** Writing – review & editing, Writing – original draft, Methodology, Investigation, Formal analysis, Conceptualization. **Amanda B. Khamas:** Writing – review & editing, Methodology, Investigation, Formal analysis, Conceptualization. **Alex Payne-Dwyer:** Writing – review & editing, Methodology, Investigation, Formal analysis. **Adam J.M. Wollman:** Writing – review & editing, Software, Formal analysis. **Kristian S. Rasmussen:** Writing – review & editing, Methodology, Investigation. **Janne K. Klitgaard:** Writing – review & editing, Methodology, Conceptualization. **Birgitte Kallipolitis:** Writing – review & editing, Methodology, Conceptualization. **Mark C. Leake:** Writing – review & editing, Supervision, Software, Resources, Methodology, Funding acquisition, Conceptualization. **Rikke L. Meyer:** Writing – review & editing, Supervision, Resources, Methodology, Funding acquisition, Conceptualization.

## Declaration of competing interest

The authors declare the following financial interests/personal relationships which may be considered as potential competing interests: Mark Leake reports financial support was provided by The 10.13039/501100000275Leverhulme Trust. Mark Leake reports financial support was provided by 10.13039/501100000266Engineering and Physical Sciences Research Council. Mark Leake reports financial support was provided by 10.13039/501100000268Biotechnology and Biological Sciences Research Council. Dominique Evans reports financial support was provided by 10.13039/100007605Aarhus University. Rikke Louise Meyer reports financial support was provided by 10.13039/501100002808Carlsberg Foundation. If there are other authors, they declare that they have no known competing financial interests or personal relationships that could have appeared to influence the work reported in this paper.

## Data Availability

Data will be made available on request.
