# Supplementary material for: Cooperation between coagulase and von willebrand factor binding protein in Staphylococcus aureus fibrin pseudocapsule formation
Source: Biofilm. 2024 Oct 23;8:100233. doi: 10.1016/j.bioflm.2024.100233 (PMC11564979; doi:10.1016/j.bioflm.2024.100233)
Supplement: Multimedia component 1 [file mmc1.docx]

**Supplementary video 1 Fibrin formation in a wildtype *S. aureus* biofilm.** Time-lapse confocal microscopy video of fibrin (red) forming in an *S. aureus* (cyan) biofilm growing in medium supplemented with 50 % human plasma. The biofilm forms cell surface-associated pseudocapsules as well as fibrin in the extended biofilm matrix. Images were acquired once every 10 min for a total time for 160 min. The biofilm was incubated at 37 °C on a microscope stage-top incubator throughout. Bacteria were visualised by gfp expression and fibrin by the addition of Alexa Fluor-647 labelled fibrinogen to the biofilm growth medium.

**Supplementary video 2 Fibrin formation in *S. aureus* that lacks Coa.** Time-lapse confocal microscopy video of fibrin (red) forming in an *S. aureus* (cyan) biofilm that lacks Coa growing in medium supplemented with 50 % human plasma. Pseudocapsules do not form in this biofilm. Images were acquired once every 10 min for a total time for 160 min. The biofilm was incubated at 37 °C on a microscope stage-top incubator throughout. Bacteria were visualised by gfp expression and fibrin by the addition of Alexa Fluor-647 labelled fibrinogen to the biofilm growth medium.

**Supplementary video 3 Fibrin formation in *S. aureus* that lacks vWbp.** Time-lapse confocal microscopy video of fibrin (red) forming in an *S. aureus* (cyan) biofilm that lacks vWbp growing in medium supplemented with 50 % human plasma. Pseudocapsules do form in this biofilm. Images were acquired once every 10 min for a total time for 160 min. The biofilm was incubated at 37 °C on a microscope stage-top incubator throughout. Bacteria were visualised by gfp expression and fibrin by the addition of Alexa Fluor-647 labelled fibrinogen to the biofilm growth medium.
