# Supplementary material for: Cooperation between coagulase and von willebrand factor binding protein in Staphylococcus aureus fibrin pseudocapsule formation
Source: Biofilm. 2024 Oct 23;8:100233. doi: 10.1016/j.bioflm.2024.100233 (PMC11564979; doi:10.1016/j.bioflm.2024.100233)
Supplement: Multimedia component 2 [file mmc2.docx]

**Cooperation between Coagulase and von Willebrand factor binding protein in *Staphylococcus aureus* fibrin pseudocapsule formation – supplementary information**

Dominique C. S. Evans^1,2^, Amanda B. Khamas^2^, Alex Payne-Dwyer^1,3^, Adam J. M. Wollman^1,3,4^, Kristian S. Rasmussen^5^, Janne K. Klitgaard^5^, Birgitte Kallipolitis^5^, Mark C. Leake^1,3^, Rikke L. Meyer^2^

^1^ School of Physics, Engineering and Technology, University of York, York, UK

^2^ Interdisciplinary Nanoscience Centre, Aarhus University, Aarhus, Denmark

^3^ Department of Biology, University of York, York, UK

^4^ Current address: Biosciences Institute, Newcastle University, NE1 7RU, UK

^5^ Department of Biochemistry and Molecular Biology, University of Southern Denmark, Odense, Denmark.

**S1. Evaluating *coa* and *vwbp* deletion mutants**

**S1.1 Methods**

The ability of the Δ*coa*, Δ*vwbp*, and Δ*coa*Δ*vwbp* mutants to coagulate whole human blood was compared to the wildtype. Whole blood was chosen because it is the natural environment of *S. aureus* during a bloodstream infection.

Whole blood was collected from a healthy individual into heparin coated tubes. Overnight cultures of the mutants and wildtype were adjusted to OD_600_ 0.1 in PBS and were then diluted 20 x into 1.5 ml whole blood. The tubes containing bacteria and blood were incubated for 48 h at 37 °C and afterwards were inspected visually to assess coagulation.

**S1.2 *coa* and *vwbp* deletion mutants coagulate human blood**

The wildtype and Δ*coa* mutant coagulated whole blood, which confirms that vWbp can trigger coagulation on its own. The Δ*vwbp* mutant partially coagulated whole blood, demonstrating that Coa can trigger coagulation on its own, but that Coa alone was insufficient to coagulate all the blood on its own. The Δ*coa*Δ*vwbp* double mutant did not coagulate whole blood, which confirms that coagulation occurred due to Coa and vWbp alone.


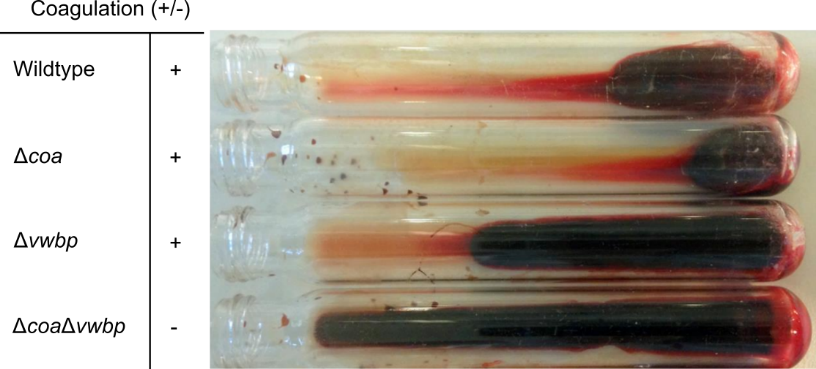


**Figure S1.1 *S. aureus* wildtype and deletion mutants coagulate human whole blood.** Coagulation assay of *S. aureus* wildtype and mutants Δ*coa*, Δ*vwbp*, and Δ*coa*Δ*vwbp* in whole human blood. Coagulation was assessed after 48 h of incubation at 37 °C. The wildtype and Δ*coa* mutant fully coagulated blood, the Δ*vwbp* mutant partially coagulated blood, and the Δ*coa*Δ*vwbp* double mutant did not coagulate blood at all.

**S2. Evaluating functionality of Coa:SNAP and vWbp:CLIP fusion proteins**

**S2.1 Methods**

The phenotype of SNAP and CLIP modified bacteria were assessed via coagulation assays and confocal laser scanning microscopy (CLSM). For the coagulation assays, overnight cultures of the mutant and its parental strain were diluted to OD_600_ 0.5 in 1 ml of 1:6 heparin stabilised human plasma in 0.85 % NaCl in sterile glass tubes and incubated for 4 hours at 37 °C with no shaking. Coagulation was assessed by tilting the tubes after 4 hours. Then the tubes were left to incubate at room temperature overnight, and the coagulation assessed again after 18 more hours. A negative control with no bacteria was also included.

For CLSM imaging, microwells (µ Slide 8 well, IBIDI, 80826) were preconditioned by incubating with 180 µl mBHI supplemented with 50 % human heparin stabilised plasma, 0.4 µg/ml Alexa Fluor 647 conjugated fibrinogen, and 1 µM SYTO 41 for 30 minutes at 37 °C. mBHI is modified BHI that contains 2.1 mM CaCl_2_ and 0.4 mM MgCl to mimic physiological conditions. The microwells were inoculated with bacteria to a final volume of 200 ul and OD_600_ 0.5 and incubated at 37 °C for 2 hours. The liquid over the biofilms was replaced with 200 ul fresh mBHI containing 50 % plasma, fluorescent fibrinogen, and SYTO 41, and incubated overnight. Samples were imaged using CLSM (LSM700, Zeiss) with 10 mW 488 nm, 5 mW 639 nm, and 5 mW 405 nm wavelength lasers operating at 2% power and a Plan-Apochromat 63x/1.40 oil immersion objective lens.

**S2.2 Coa:SNAP and vWbp:CLIP modified bacteria coagulate human plasma**

Coa and vWbp both hijack the host coagulation cascade to produce a fibrin clot. Therefore, the ability of the modified proteins to coagulate human plasma was compared to the native proteins to assess whether the proteins functioned correctly when fused to SNAP/CLIP. Bacteria containing either modified or unmodified Coa/vWbp were incubated with human plasma and the coagulation was assessed after 4 and 24 hours. All strains coagulated plasma by 4 hours, apart from a double mutant lacking both Coa and vWbp (Table S2, Figure S2.1), which confirmed that coagulation occurred due to Coa and vWbp alone. Therefore, Coa and vWbp could still function to coagulate plasma when fused to SNAP and CLIP.


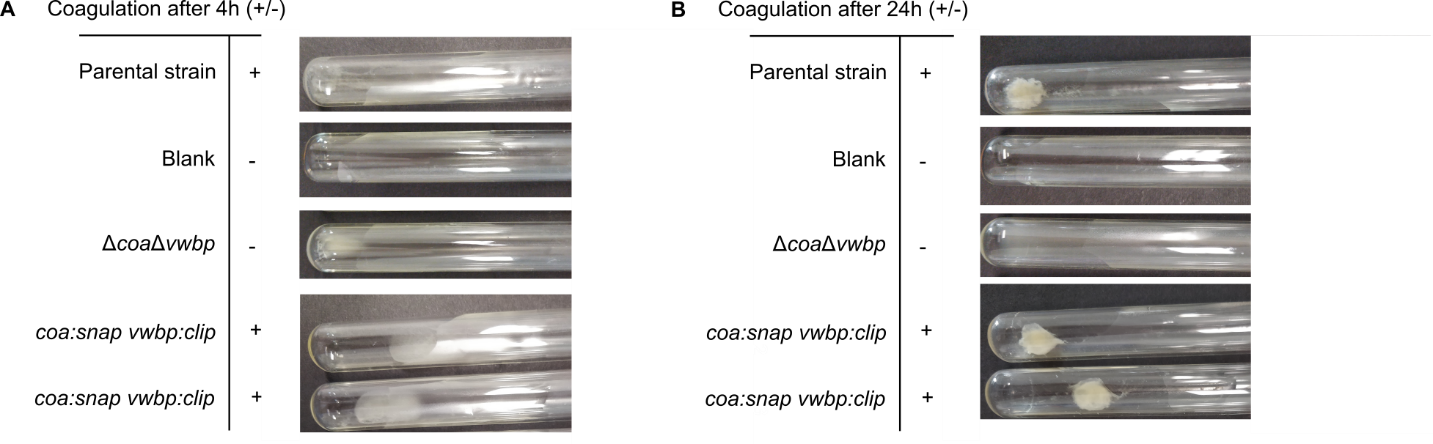


**Figure S2.1 *S. aureus* producing Coa:SNAP and vWbp:CLIP coagulate human plasma the same as the parental strains.** Coagulation assays of *S. aureus* strains incubated at 37 °C with human plasma for **a)** 4 h and **b)** 24 h. The tubes contain either the parental, unmodified wildtype strain, a blank that contains plasma but no bacteria, the double mutant which lacks both coagulases, or two clones of *S. aureus* modified with Coa:SNAP and vWbp:CLIP.

**Table S2** Coagulation of *S. aureus* strains with modified and unmodified Coa and vWbp.

|  | **Coagulation (+/-)** | | | |
| --- | --- | --- | --- | --- |
|  | **vWbp:CLIP & Coa:SNAP** | **Parental strain** | | **Blank** |
|  | **wt** | **wt** | **Δ*coa*Δ*vwbp*** |  |
| **4 h** | + | + | - | - |
| **24h** | + | + | - | - |

**S2.3 Coa:SNAP and vWbp:CLIP modified bacteria produce biofilms with the same phenotype as the parental strains**

Coa and vWbp cause a fibrin network to form in the biofilm extracellular matrix. As an additional check to verify that the fusion proteins functioned as expected, the phenotype of the fibrin matrix was assessed via CLSM in bacteria with modified and unmodified Coa/vWbp. The fibrin networks of all modified strains resembled the parental strains (Figure S2.2). There were no clear differences between the modified and parental strains, except for a large increase in fibrin signal in the parental wildtype, which could have been due to a pipetting or mixing error resulting in an increased concentration of fluorescent fibrinogen during sample preparation since this increase in signal was not present in any of the other parental strains. Overall, the modified bacteria produced a fibrin network as expected and the fusion proteins functioned correctly.

**
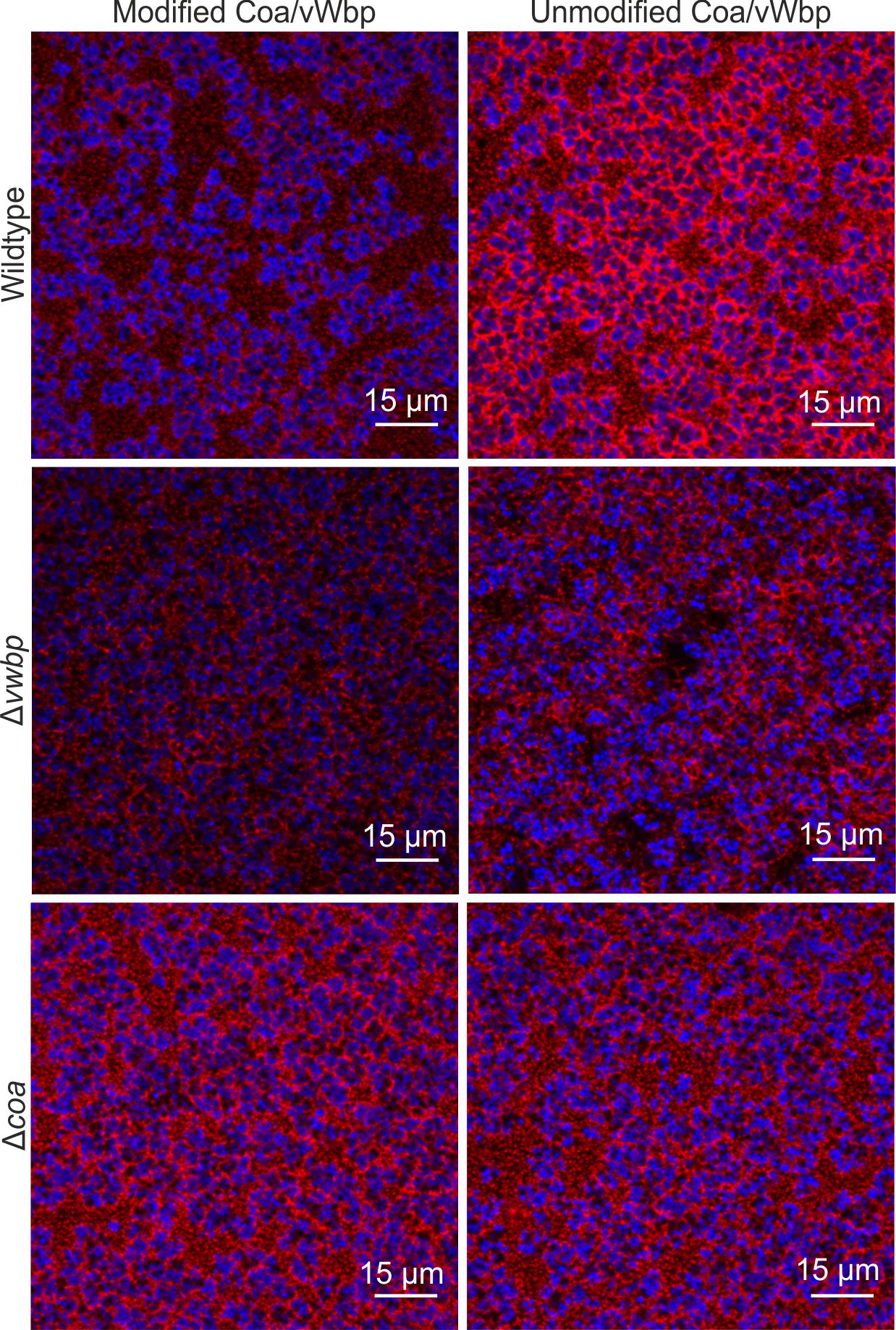
**

Figure S2.2 *S. aureus* producing Coa:SNAP and vWbp:CLIP produce biofilms containing a fibrin matrix the same as the parental wildtype strain. CLSM images of *S. aureus* biofilms formed with modified and unmodified Coa/vWbp. Cells are labelled with the DNA-binding stain SYTO 41 (blue) and fibrin is labelled by incorporation of Alexa Fluor 647-conjugated fibrinogen (red).

**S3. *S. aureus* produce more Coa and vWbp when grown with host factors**

We investigated two conditions that may affect the production of Coa and vWbp: growth phase and presence of host factors. Relative differences in the expression of Coa and vWbp was assessed by how quickly coagulation occurred when cell culture supernatants were transferred to human plasma. Further production of Coa and vWbp after transferring to human plasma was inhibited by the addition of chloramphenicol. The assay therefore reflected the activity of Coa and vWbp synthesised during the incubation taking place prior to the coagulation test. We tested the coagulation ability of *S. aureus* cells and filtered supernatants from *S. aureus*, *S. aureus* Δ*vwbp*, *S. aureus* Δ*coa*, *S. aureus* Δ*coa*Δ*vwbp*, and *S. xylosus* cultures. Bacteria were grown to either exponential or stationary phase, and in the presence or absence of human serum.

Results are summarised in Table S3. Coagulation time was faster for the mutants lacking Coa or vWbp when grown to exponential phase. The wild type consistently coagulated after 24 hours but not by 4 hours when grown in the absence of host factors, and Δ*vwbp* and Δ*coa* only coagulated by 24 hours when grown to exponential phase, so Coa and vWbp levels were higher when grown to exponential phase. Addition of host factors enhanced Coa production and accelerated coagulation time to 4 hours in Δ*vwbp* in both exponential and stationary phase cultures. Host factors also enhanced vWbp production when grown to stationary phase. We therefore chose to prepare cultures for subsequent experiments by growing to mid-exponential phase in BHI supplemented by 5 % serum.

**Table S3.** Tube coagulation tests for human plasma with filtered supernatant from *S. aureus*, *S. aureus* deletion mutants, and coagulase negative *S. xylosus* cultures. Cultures were grown to exponential or stationary phase in either plain BHI or BHI supplemented with 5 % human serum, and coagulation assessed after 4 and 24 hours.

|  | **Coagulation (+/-) (no serum, exponential phase)** | | **Coagulation (+/-) (5 % serum, exponential phase)** | | **Coagulation (+/-) (no serum, stationary phase)** | | **Coagulation (+/-) (5 % serum, stationary phase)** | |
| --- | --- | --- | --- | --- | --- | --- | --- | --- |
|  | **4h** | **24h** | **4h** | **24h** | **4h** | **24h** | **4h** | **24h** |
| **Wild type** | **-** | **+** | **+** | **+** | **-** | **+** | **+** | **+** |
| **Δ*vwbp*** | **-** | **+** | **+** | **+** | **-** | **-** | **+** | **+** |
| **Δ*coa*** | **-** | **+** | **-** | **+** | **-** | **-** | **-** | **+** |
| **Δ*coa*Δ*vwbp*** | **-** | **-** | **-** | **-** | **-** | **-** | **-** | **-** |
| ***S. xylosus*** | **-** | **-** | **-** | **-** | **-** | **-** | **-** | **-** |
